# Mobile applications for the sport and exercise nutritionist: a narrative review

**DOI:** 10.1186/s13102-022-00419-z

**Published:** 2022-02-22

**Authors:** Daniel J. Peart, Marc A. Briggs, Matthew P. Shaw

**Affiliations:** 1 Department of Sport, Exercise and Rehabilitation, Northumbria University, Newcastle-upon-Tyne, UK; 2 Sports, Physical Activity and Food, Western Norway University of Applied Sciences, Sogndal, Norway

**Keywords:** Diet analysis, Body composition, Education, Apps, Smartphone

## Abstract

Mobile technology is widespread in modern society, and the applications (apps) that they run can serve various purposes. Features such as portability, ease of communication, storage, and relative low cost may make such technology attractive to practitioners in several fields. This review provides a critical narrative on the existing literature for apps relevant to the field of sport and exercise nutrition. Three main areas are discussed: (1) dietary analysis of athletes, (2) nutrition education for athletes, (3) estimating body composition. The key purpose of the review was to identify what literature is available, in what areas apps may have a benefit over traditional methods, and considerations that practitioners should make before they implement apps into their practice or recommend their use to coaches and athletes.

## Background

The ubiquity of smartphones has led to developments in easily accessible methods of collecting, storing, and sharing information via software applications and hardware components typical to most devices. Specifically, within sport and exercise science, smartphone devices can be used to collect variables such as heart rate, jump height, barbell velocity, and distances covered, albeit with differing levels of reliability and validity dependent on the app selected [1]. When their validity is simple to determine, sport and exercise practitioners are more likely to use such smartphone applications (apps) in their practice [2]. Therefore, it is important to regularly review the body of evidence to inform practice as technology advances and more software applications become commercially available for public use.

The appetite to utilise such smartphone technology is also apparent in dietetics, with an international survey by Chen et al. [3] finding that 62% of 570 dietitians used nutrition apps in their own practice, and 84% recommended them to their patients. The most common uses were for sharing information resources, patient self-monitoring, extra patient support, and dietary analysis. These common uses have some evidence to support their use, such as apps for patient education in areas such as blood glucose management [4], and apps with photo-based methods of dietary analysis [5]. Apps have been used for similar means by sport and exercise dietitians and nutritionists [6]. However, it cannot be assumed that the dietetic or nutrition research evidence in a clinical field can transfer to sport and exercise nutrition, as athletes are a unique population that whilst healthy, are at risk of energy and nutrient deficiencies; particularly young athletes [7].

The purpose of this paper is to review the current evidence base for using smartphone technology in sport and exercise nutrition practice. The review will focus on three areas of practice relevant to the field: (1) dietary analysis of athletes, (2) nutrition *counselling and* education of athletes, (3) estimating body composition.

## Dietary analysis

Traditional methods of assessing energy intake have been questioned. Whilst diet recalls provide a quantitative assessment of energy intake, recollection periods and accuracy are usually limited [8]. Furthermore, food-frequency questionnaires and diet history methods may offer greater insight into habitual energy intake but have been criticised for their seasonality and lack of detail for individual quantification [8]. Subsequently, food diaries were identified as a more accurate method of nutritional assessment, involving the weighing or estimated quantification of food and drink items [9]. However, it is important to acknowledge that food diaries may also be susceptible to reporting bias [8]. Most of the evidence suggests an underestimation of energy intake when using self-reported weighed or estimated food diaries, of ~ 11–27% [10–13], highlighting the difficulties of providing accurate analysis and intervention. Alternative methods have been developed such as the combined method of self-report, weighed food diary and 24 h recall, which demonstrated a low random error (3.1%) between methods, and although a statistically significant under-reporting was observed (88 kcal day^−1^), the magnitude of this bias was small [14]. Whilst there is evidence of improved accuracy with some self-reported methods, this does not eradicate the participant burden associated with traditional methods of energy intake collection. Furthermore, the labour-intensive nature of dietary analysis by trained nutritionists can also be prone to error [15].

Advances in technology and the prevalence of smartphones in society, present opportunities to reduce some of the challenges, synonymous with traditional self-reporting methods. The plethora of mobile apps for tracking energy intake enable real-time recording, which can be simultaneously analysed and compared to pre-determined intervention goals or recommended intake values. Whilst apps share the portability benefits of traditional dietary records, the additional features of automated time/date-stamped recording of items alongside features such as barcode scanning and image taking provide greater objectivity [16]. However, current reviews acknowledge the algorithms required for image-assisted apps are still in their infancy, with practitioners and researchers predominantly opting for apps which utilise more textual food input methods [17, 18].

The concept of apps tracking energy intake is not necessarily new, the underpinning principles align with traditional methods of dietary record, however the technology utilised in these apps alter the method in which dietary data is collected. It is therefore important to investigate the validation of these apps, prior to the implementation into both research and practice to ensure accurate and reliable data is being collected. With the dominance of smartphone usage within society, the concept of using photography-based dietary assessment has recently been developed in smartphone apps. One such app (Snap-N-Send), was found to be valid and reliable when compared to researcher-observed methods in free-living environments [19]. Despite a reported significant small mean bias of 179 kcal day^−1^ it was concluded that the Snap-N-Send app was an accurate method for assessing energy intake in elite adolescent athletes at the group level [19]. In a subsequent study by Costello et al. [20], the dietary assessment accuracy of Snap-N-Send was compared against doubly labelled water with elite adolescent athletes. Although a small systematic bias was again highlighted (50–122 kcal day^−1^) at the group level, questions remain, as Snap-N-Send demonstrated large random error, highlighting the reduced measurement accuracy at an individual level.

MyFitnessPal (MFP) is a free smartphone app and considered one of the most popular apps for sports nutritionists [6]. MFP calculates energy intake using a relatively user-friendly interface composing a database of over 3 million food and drink items, with the capacity to enter any additional items manually. In a recent study, Teixeira et al. [21] investigated the validity of the MFP app, using paper-based food records as a reference method. Even though systematic errors were evident, total energy and fibre demonstrated moderate correlations with food records, showing good relative validity. However, further analysis indicates MFP tends to underestimate individual nutrients, and is not recommended for research purposes, despite users preferring this method of dietary assessment. Explanations of such inaccuracies were purported to be due to database inaccuracies [21]. Another app (Bridge2U) has also been developed as an electronic food log to reduce researcher burden and cost due to its real-time data entry and detection. However, the reporting accuracy has been criticised. The Bridge2U app demonstrated significant underreporting of energy intake when compared to 24 h recall [22]. Whilst group level data suggests the Bridge2U app is a valid tool, the wide variability of subject data entry undermines use for individual assessment. To improve the limitations of app usage for accurate dietary assessment, the newly developed PIQNIQ app incorporates features such as text entry with dropdown menus and a comprehensive database, as well as portion size selector combined with slider aid for *visual* estimation of food and drink items. In a study investigating the accuracy of the PIQNIQ app results indicate that, with the exception of added sugars and total fats, macro and micronutrient profiles were comparable to interviewer administered multiple-pass 24 h recalls [23]. Findings are encouraging, suggesting apps which are carefully designed to incorporate user-friendly portion size selection tools combined with image assistance, and links to comprehensive databases, may produce similar accuracy as traditional validated methods, whilst reducing the burden of such methods.

In one of the first reviews investigating the feasibility and validity of smartphone apps, specifically designed for dietary data collection, Sharp et al. [24] concluded that such apps provided only similar validity and reliability when compared to traditional methods, failing to demonstrate significant improvements. It is important to note that this review included apps developed between 2001 and 2013 and may now not account for any technological developments in this field. Furthermore, validation studies included in the review were criticised for the limited duration of collection periods, presenting challenges when attempting to quantify habitual intake, whilst also implementing insufficient rigour during statistical analysis [24]. Despite the lack of progression in accuracy, apps were highlighted as the preferred option partly due to the reduction in both researcher and participant burden, cost reduction and real time data coding [24]. Additionally, the pragmatic approach of real-time communication with participants, providing prompts for more detailed info, allows the correction of ambiguous entries and enables individualised recommendations during intervention studies.

In a more recent review, Zhang et al. [18] conducted a systematic review and meta-analysis on validation studies of dietary apps. Sample sizes ranged from 18 to 362, predominantly including young adults over a 2–7 days collection period, within real-life settings. Apps used within these validation studies included MyFitnessPal, Diet-A, EVIDENT II, Eat and Track, FoodNow, Nutrabem, e-CA, MyMealMate, e-DIA, BENECA and Research Food Diary. Findings highlighted that all apps underestimated energy intake when compared to their reference methods, with a pooled effect of − 202 kcal day^−1^ (95% CI − 319 to 85 kcal day^−1^) [18]. Wang et al. [25] proposed a difference in energy intake of 110–165 kcal day^−1^ to be clinically meaningful in a weight loss context; lower than the pooled effect of the underestimation of energy intake when compared to their respective reference method identified in the meta-analysis (− 202 kcal day^−1^). This suggests the lack of accuracy would likely impact on energy balance if used in applied and/or research contexts to design recommendations/interventions. Further analysis of the findings suggest unintentional or intentional under-reporting bias was similar between apps and traditional methods. However the current review excluded apps which allowed image-based analysis or automated prompts to promote greater accuracy, a feature which has been shown to improve objectivity and accuracy of energy intake [26]. It is important to acknowledge that 11 out of the 14 studies included within the meta-analysis used 24 h recalls as the reference method. It is widely accepted in the literature that 24 h recalls are prone to significant subject bias and have limited application for habitual energy intake [27], which somewhat undermines the validation findings. Subsequently, absolute validity was unclear and not reported. The inclusion of objective reference methods such as doubly labelled water could provide a more insightful understanding of validity and provide greater evidence of the inclusion of app-based energy intake collection methods in the future. Interestingly, the studies included within the review which used objective tools as energy intake reference methods (accelerometery) demonstrated more favourable results [16, 28, 29]. Reliability between estimated energy intake and measured energy expenditure was high (ICC, 95% CI 0.75, 0.61–0.84) suggesting the FoodNow app could be used at the group level as a suitable alternative [16].

It is important to acknowledge that whilst this paper attempts to discuss the practicalities of smartphone app usage in athletic populations, research using such participants are limited. Subsequently research has been drawn from a range of adolescent and adult populations. However, there is evidence to suggest that subject bias with regards to over and under-reporting of energy intake is prevalent in both athletic and non-athletic populations with similar degrees of quantification [30], suggesting extrapolation of non-athletic data may still be relevant. It is clear to see that apps have potential benefits over their traditional self-reporting counterparts by utilising smartphones, which have become an increasingly dominant aspect of modern society. The portability, automated-prompts, analysis of nutrients and objectivity of incorporating images, all aid in the reduction of participant and researcher burden. However, current validation studies provide equivocal evidence of the accuracy of energy intake assessment. Further research is warranted to explore validation methods utilising objective reference methods to determine whether the advancement of technology can progress the field of energy intake assessment.

## Nutrition counselling and education

*Education does not always result in behaviour change, but there are examples* of online interventions that have been shown to be effective to instigate behaviour change [31], and a recent systematic review identified that apps can be effective interventions for nutrition behaviour [32]. Furthermore, this type of intervention has been adopted in practice, with one study reporting that almost half (46%) of 570 responding dietitians used apps as an education resource for their patients [3]. However, this past work on clinical populations may not necessarily transfer to a sporting population, and athletes are a unique population as, whilst healthy, are still at risk of nutritional imbalances [33, 34]. This may be particularly true of young athletes [7], who are a population who may respond well to online interventions, with Zuniga et al. [35] stating that young athletes are interested in potential application-based interventions. Development work exists that reports the potential of sport nutrition education apps, but they do not evaluate their use in practice [36, 37]. One 8-week case study from Curtis et al. [38] assessed the dietary intake of two 19-year-old female rugby union players. One player used the MealLogger app to submit photos of their meals, on which they received feedback related to suitability, composition, and timing, whereas the other player received usual support (fortnightly correspondence). Both players increased their total energy intake, but the player using the app did so by increasing carbohydrate and protein intake, and the other did so by increasing fat and reducing carbohydrate and protein intake. It is not clear if these changes were meaningful or within typical daily variance for the individual athletes, and it is difficult to extrapolate the findings to a larger population, as the authors themselves acknowledge. However, of interest is that the intervention allowed a more frequent daily, as opposed to fortnightly, communication between the practitioner and athlete, which may have wider reaching benefits in terms of building a professional relationship.

Simpson et al. [39] used the same MealLogger app to provide nutritional feedback to a group of seventeen 18–20 year-old male field hockey players for 6 weeks. Players logged images of their food three times per week to receive practitioner feedback, and received an information document on a different nutrition topic weekly through the app. In a single group pre-post design, the athletes demonstrated increased nutrition knowledge as measured by the ‘Questionnaire of Nutrition Knowledge’, and an increased perceived knowledge and confidence. They all perceived the intervention to have had some influence on their behaviour, particularly the element of being able to view each other’s meals. The outcome measures employed in this study make it difficult to evaluate the efficacy of the intervention, as an improved knowledge does not necessarily translate to a change in behaviour, even if that is what the athletes perceived. The design of the study also means that it is unknown whether the MealLogger app would have outperformed other more traditional interventions. However, of note is that before the study none of the athletes said that they would prefer to receive advice from a sports dietitian, whereas after the study this increased to 82%. Therefore, like the observations of Curtis et al. [38], whilst the study could not provide strong evidence for behaviour change, the app based intervention did provide an opportunity for improving the athlete-practitioner relationship.

Unlike Simpson et al. [39], Heikkilä et al. [40] did design a study to investigate if increased nutrition knowledge through an app-based intervention translated to a change in behaviour. They also implemented an alternative education intervention as a comparison group. Seventy-nine endurance athletes aged 18 ± 1.4 years received three 90-min sessions on a fortnightly basis, completing a nutrition knowledge questionnaire [41] at weeks 0, 5 (post intervention), and 17 (12 week follow up). For one half of the group this was the sole intervention (EDU), whereas the others also logged images of their meals on MealLogger for 4 days after each 90-min session (EDU + APP). Those in the EDU + APP group were asked to upload a commentary alongside their images with a different focus each time ([1] meal timing and fluids, [2] healthy eating, [3] variety and vitamin D). Nutrition knowledge increased in both groups at week 5 and remained elevated above baseline at week 17, with no difference between the groups. However, despite this macronutrient and energy intake did not increase significantly in either group, and remained below pre-determined recommendations. Therefore, it was concluded that the app intervention improved knowledge but was not better than the traditional sessions alone, and neither intervention had an influence on athlete behaviour.

A pair of studies from Budiono et al. describe the use of an app called Nutriatlet with Martial Artists. The first study describes how the app uses a food unit substitution method to help athletes attain an individual goal intake [42]. The example provided was an athlete requiring 3500 kcal would consume 11 units of carbohydrate, 4 units of animal protein, 4 units of vegetable protein, 4 units of vegetable, 4 units of fruit, 2 units of milk, 3 units of oil, and 6 units of sugar. However, it is unclear how the ratio of units was determined, and how the equivalence of foods within each category were checked. Nevertheless, athletes using the app reached their goal intake within 6.3 weeks, 2 weeks faster than a control group. However, it was not described what education the control group received. A second study aimed to use the app to support Martial Artists lose weight without sacrificing good nutritional practice [43]. The app was used in a similar fashion in terms of recommending units of food types, but in this paper, it is better described how it was managed, with athletes receiving food from a central service within the facility. It was then up to athletes to use the apps to dictate portion sizes of the foods on offer. At the end of the 30 days they observed increased energy intake (from 64 to 83% of target), reduced body fat, and maintained body mass index (BMI). This combination of results is promising, as for body fat to decrease despite increased energy intake and a stable BMI, this means that optimum protein was being consumed [44]. Therefore, with an increase in energy expenditure this intervention may help athletes on progressive weight loss plans such as those described in past case studies [45, 46].

## Body composition

Photogrammetry is the extraction of the geometry of a structure, e.g. human body, using photographs or digital images [47], thus providing various lengths and widths. Using 2-and-3-dimensional photographic images for anthropometrical measures has existed for multiple decades [48] and the existing literature has demonstrated reliability and validity in various contexts such as using volume-to-mass conversion factors to estimate body mass [49] and identification of craniofacial landmarks [50]. This sub-discipline of photogrammetry, “digital anthropometry” [51], has extended beyond surface measurements to provide estimation of tissue composition. As highlighted previously in a wider review of smartphone applications in sport and exercise science [1], there remains a limited amount of digital anthropometry apps that are commercially-available. Engineers and software developers have utilised smartphone hardware in conjunction with custom made software to demonstrate reliable estimates of body fat percentage (%BF) [52, 53]. However, the software developed in this literature is not commercially available via App store or Google Play. For smartphone apps that are available to the public, and therefore practicing nutritionists, the results are mixed. For example, LeanScreen (Postureco, Trinity, FL, USA) is a smartphone app that estimates percentage body fat by digitizing a series of girths within 2-dimensional (2D) photographs. This reliability of the app has been supported, with inter- and intrareliability coefficients of ≥ 0.99 in a study from MacDonald et al. [54], and an intratester coefficient of 0.974 in another study [55]. Of note in the latter study though is that typical error of measurement (TEM), a measure of within-subject variation calculated as the standard deviation of repeated measurements, was higher in the app (TEM = 1.6%BF) when compared to skinfold calipers (TEM = 0.37%BF) and bioelectrical impedance analysis (TEM = 0.23%BF). The app also produced a higher coefficient of variation (CV = 6.4%BF) compared to skinfold callipers (CV = 1.1%BF) and bioelectrical impedance analysis (CV = 0.7%BF).

In terms of validity, the app had a greater bias and underestimation of bodyfat percentage when compared to dual energy X-ray absorptiometry (DXA) (− 3.3%BF) [54], and air displacement plethysmography (− 2%BF) [56]. Neufeld et al. [57] found more promising results for the LeanScreen app, demonstrating acceptable convergent validity, and therefore high agreement, with both bioelectrical impedance analysis (r, 95% CI 0.82, 0.77–0.87) and skinfold callipers (r. 95% CI 0.83, 0.78–0.86). Average measurement bias [95% CI] was 1.8 [1.2–2.4] and 0.5 [0.0–1.0] compared to bioelectrical impedance and skinfold measurements respectively. However they did not take any measurements using DXA or plethysmography so it is difficult to compare the findings to the work of MacDonald et al. [54] and Wagner et al. [56]. The large sample size of 240 participants can give confidence in the findings, but practitioners should note some features of the study to inform their decision on implementing it in their practice. Firstly, the app was only tested on a relatively narrow range of body sizes (23.3 ± 2.8 kg m^2^) so its accuracy outside of this is unknown, and secondly all participants wore tight fitted clothing so this would need to be matched in practice. Finally, whilst the measurements may be like field-based alternatives such as bioelectrical impedance analysis and skinfold measures, the study did not compare the app to a laboratory method, so criterion validity was not assessed.

More recently Tian et al. [58] describe a similar method of using 2D images to predict body composition, showing that a single smartphone-captured image can provide total and regional body composition estimates comparable to DXA scans. However, like Choi et al. [52] and Farina et al. [53], the investigation by Tian et al. [58] did not examine an application that is currently available to the general public. This technology is relatively new [54, 56] and Wagner et al. [56] suggests there may be issues with the software’s (LeanScreen) predictive algorithm, as opposed to a lack of validity in 2D digital anthropometry. 3D optical imaging appears to be a more reliable digital anthropometry technology for estimating body composition [51], demonstrating strong correlations with more expensive advanced imaging equipment e.g. DXA [59]. Although, interestingly, Neufeld et al. [57] found that average bias increased from 1.8%BF to 4.8%BF compared to bioelectrical impedance analysis and from 0.5%BF to 3.4%BF compared to skinfold measurements when a 3D photonic scanner was attached to the device (Structure Sensor; Occipital, Inc., San Francisco, CA, USA). Without comparison to a criterion measure it is unclear if this difference represents an improvement or reduction in measurement accuracy, so future work is warranted using 3D imaging techniques using mobile technology.

## Future perspectives

Most of the research examining the use of mobile apps for dietary analysis has been conducted in a non-athletic population, but as acknowledged in the relevant previous section, similar levels of reporting bias have been reported for other methods of energy intake estimation between athletic and non-athletic groups. However future work should still investigate athletic populations to confirm that this is the case for mobile apps too. Other factors relevant for athletes that may need to be considered in future is the sensitivity to estimate micronutrient intake and amino acid profiles, and the ability to identify changing eating patterns throughout a periodised plan. Another area of study could be the added functionality of some apps to allow energy expenditure estimation, and what implications this may have on athlete behaviour if they can see an estimated energy balance over a period of time. Research in apps for nutrition education research has investigated the role of communication and food selection on behaviour, but future work may wish to consider employing food skills advice within the app in case that is a barrier to education implementation in a home-setting. Body composition research is still in its infancy and the validity and reliability of mobile apps have been tested in a relatively narrow range of participants, so the applicability of the findings to the extremes of body sizes witnessed in sport are unknown. The sensitivity of these apps to map changes in body composition over different points of a competitive season also needs investigation.

## Summary and conclusions

**Fig. 1:**
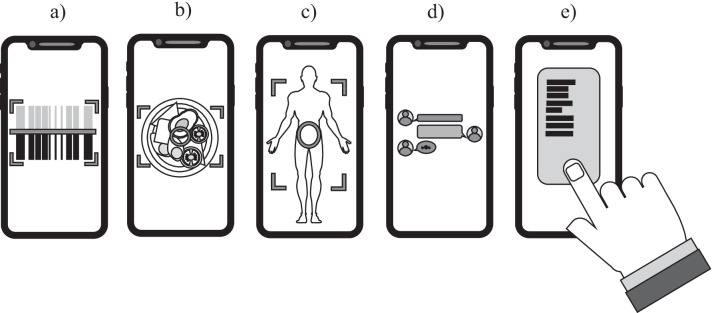
An illustration of the relevant functionality of mobile devices for the topics covered in this review: **a** barcode scanning for dietary analysis, **b** image capturing for dietary analysis, **c** image capturing for body composition, **d** communication between peers, nutritionist, coaches etc., **e** manual data entry

This review focused on the potential of mobile apps to support sport and exercise nutrition practice in three main areas: (1) dietary analysis, (2) nutrition education, and (3) body composition estimation. The functionality of mobile devices related to these aspects are summarised in Fig. 1, and the potential benefits of this technology, and key points to consider before using it, have been summarised in Table 1. In addition to the assumed benefits associated with cost, ease of access and portability, beneficial features related to data input and coding have been reported for dietary analysis, and the capability for image capture and automated prompts were shared benefits for dietary analysis and nutrition education. Connectivity between athletes and practitioners, and athletes and other athletes, was an advantage for mobile apps as an education tool. This was particularly true for young athletic populations, although there is limited research in other age groups to say that they do not work as well in older athletes. Mobile apps also have some limitations, for example dietary intake can be underestimated if due consideration is not given to the database that the app derives its data from, the portion size interface, and the ease of use for participant adherence. In addition, mobile apps are typically shown to match rather than better traditional tools for dietary analysis and nutrition education. In terms of body composition measurement, existing studies differ in design making a firm conclusion difficult. Future work is required to compare both an app and its field based alternatives with a criterion measure across a range of body sizes so the practitioner can make an informed decision about which field based measurement they implement. Therefore, this review does not present an argument for mobile apps to become recommended practice in place of traditional methods in the field of sport and exercise nutrition. However, practitioners can use this paper to make an informed choice if they choose to try this technology, and its continued use should be judged based on personal preference and what works for the athlete-practitioner relationship in question.

**Table 1:** A summary of the potential benefits of apps and some of the points to consider before implementation into practice

	Potential benefits of using mobile applications	Key points to consider before implementation
Dietary analysis	Barcode scanning and camera functions simplify data entry Many apps provide real time data coding The above could combine to reduce participant and practitioner burden	Apps typically underestimate energy intake compared to other methods Incorporating images and automated prompts may improve accuracy Apps may also be more accurate when they include a user friendly and intuitive portion size interface Practitioners should understand what database the app derives nutrition data from to allow comparison to past athlete data and other tools
Nutrition *counselling and* education	Facilitates efficient and regular contact between the athlete and practitioner Repeatedly found to improve athlete nutrition knowledge	Whilst effective, there is limited evidence that they are more effective than other forms of intervention to enhance nutrition knowledge. Athlete preference may be key App led increases in nutrition knowledge have not always resulted in changed nutrition behaviour. Therefore, behaviour change should not be assumed, and practitioners should continue to monitor athlete nutrition practice
Body composition estimation	Portability and cost	There is some evidence that measurement taken from the LeanScreen app may be reproducible As criterion validity is not confirmed it is recommended that practitioners take results on this app in isolation and do not use it interchangeably with other methods. Individual client variance should be determined to understand a worthwhile change

## Data Availability

Not applicable.
